# Impaired Frontal-Basal Ganglia Connectivity in Male Adolescents with Conduct Disorder

**DOI:** 10.1371/journal.pone.0145011

**Published:** 2015-12-14

**Authors:** Jibiao Zhang, Baojuan Li, Junling Gao, Huqing Shi, Xiang Wang, Yali Jiang, Qingsen Ming, Yidian Gao, Ren Ma, Shuqiao Yao

**Affiliations:** 1 Department of Psychology, School of Education, Jianghan University, Wuhan, Hubei, China; 2 Medical Psychological Institute, Second Xiangya Hospital, Central South University, Changsha, Hunan, China; 3 School of Biomedical Engineering, Fourth Military Medical University, Xi’an, Shaanxi, China; 4 Centre of Buddhist Studies, University of Hong Kong, Hong Kong, China; 5 Department of Psychology, Shanghai Normal University, Shanghai, China; 6 National Technology Institute of Psychiatry, Changsha, Hunan, China; Wake Forest School of Medicine, UNITED STATES

## Abstract

Alack of inhibition control has been found in subjects with conduct disorder (CD), but the underlying neuropathophysiology remains poorly understood. The current study investigated the different mechanism of inhibition control in adolescent-onset CD males (n = 29) and well-matched healthy controls (HCs) (n = 40) when performing a GoStop task by functional magnetic resonance images. Effective connectivity (EC) within the inhibition control network was analyzed using a stochastic dynamic causality model. We found that EC within the inhibition control network was significantly different in the CD group when compared to the HCs. Exploratory relationship analysis revealed significant negative associations between EC between the IFG and striatum and behavioral scale scores in the CD group. These results suggest for the first time that the failure of inhibition control in subjects with CD might be associated with aberrant connectivity of the frontal–basal ganglia pathways, especially between the IFG and striatum.

## Introduction

Conduct disorder (CD) is an impulse control–related disorder characterized by impulsivity, aggression toward people or animals, property destruction, deceptiveness or theft, and serious rule violation before the age of 18 years [1]. CD has been reported to occur in about 16% of preadolescents [2], and it usually co-exists with other disorders, such asattention-deficit/hyperactivity disorder (ADHD), oppositional defiant disorder (ODD), and substance abuse [3]. CD incurs a large social cost [4], as it is almost always a prognosticator of antisocial personality disorder in adulthood [5].

In past decades, an enormous amount of neuroimaging research has focused on the underlying pathophysiological mechanism of CD. Converging evidence from functional magnetic resonance imaging (fMRI) studies has pointed to dysregulation in the orbitofrontal cortex (OFC) [6–10], ventromedial prefrontal cortex (PFC) [11,12], insula [12,13], striatum [7,12,14,15] and amygdala [6,14,16] in individuals with CD relative to healthy controls (HCs) when processing emotion- and reward-related tasks. These findings have been confirmed in structural magnetic resonance imaging studies. In addition, the volumes of the PFC (including the OFC) [9,17,18], temporal cortex [17,19], amygdala [20–22], insula [20,21,23] and striatum [22,23] were found to be decreased in males with CD compared with HCs.

Alack of inhibition control has also been found to be prominent in subjects with CD [1,24,25]. For example, Dougherty et al found the CD individuals showed a lower inhibited response rates to stop trials than the HCs in the GoStop task [26], a paradigm used to measure the capacity to inhibit an initiated predominant response [27]. In a previous study, we showed that individuals with CD displayed increased impulsivity [28]. Converging evidence has suggested that the stop-signal task (SST) is a suitable experimental paradigm for the examination of motor inhibitory control in various populations [24,29]. Previous research has documented the involvement of the frontal–basal ganglia pathway, including the inferior frontal gyrus (IFG) [29–31], supplementary motor area (pre-SMA) [30–32], striatum [30,31,33], subthalamic nucleus (STN) [29–31] and thalamus [29,31,34] in “stop” networks. However, a limited number of fMRI studies to date have investigated the neurofunctional substrate of inhibitory control in CD. Notably, Rubia and colleagues (2008) found significantly reduced activation of the dorsolateral PFC, bilateral temporoparietal cortex, posterior cingulated gyrus, striatum, and thalamus in boys with CD compared with HCs when performing the modified SST [35].

With advances in analytical technology and the notion of connectivity networks, researchers recognized that the etiology of conduct problems may be not only attributable to variations within several specific brain regions, but also associated with the connections between them. Thus, further detailed research exploring the dysfunction underlying poor response inhibition in subjects with CD is needed. The aim of this study was to use fMRI to investigate differences in the neurobiology of inhibitory control in a carefully selected “pure” CD group compared with that in well-matched HC adolescents. We sought to substantiate the roles of the “stop” network in the motor inhibition task by using the stochastic dynamic causality model (DCM), a technique that measures direct (effective) connectivity (EC) among brain regions at the neuronal level [36]. We hypothesized that lower overall connection strengths between the brain regions of the “stop" network would be found in subjects with CD relative to HCs. Additionally, we investigated relationships between connection strengths and behavioral scores.

## Results

### Behavioral scores

The two groups were well matched, with no significant difference in age, IQ, socioeconomic status, depression symptomology, or anxiety severity (p > .05). SDQ and APSD total and subscale scores were significantly higher in the CD group than in the HC group (p < .05), but all were below clinical thresholds. BIS total scale and motor and unplanned subscale scores were significantly higher in the CD group than in the HC group (p < .01), indicating that subjects with CD were more impulsive than HCs (Table 1, S1 File).

**Table 1:** Demographic and clinical characteristics of study participants.

	Conduct disorder (n = 29)	Healthy control (n = 40)	*t*-value	*p*-value
Age (years)	15.14±0.92	15.48±0.78	-1.643	0.105
IQ	103.38±10.27	105.18±7.30	-0.806	0.425
SSS	6.00±1.67	6.03±1.37	-0.068	0.946
CES-D	14.17±4.04	12.37±5.31	1.530	0.131
MASC	39.97±19.99	37.47±15.84	0.569	0.571
SDQ-conduct problems	4.24±2.28	2.48±1.32	4.059	0.000**
SDQtotal	14.76±5.82	11.88±5.06	2.194	0.032*
APSD-CU traits	5.69±1.63	4.56±1.59	2.825	0.006**
APSD-impulsivity	4.72±2.39	3.39±1.87	2.528	0.014*
APSDtotal	14.76±4.10	11.69±3.26	3.357	0.001**
BIS-attention impulsivity	18.83±3.67	18.18±2.64	0.859	0.393
BIS-motor impulsivity	26.93±4.54	21.68±3.51	5.427	0.000**
BIS-unplanned impulsivity	31.93±3.98	27.50±4.28	4.370	0.000**
BIStotal	77.69±10.25	67.35±7.32	4.892	0.000**

Data are presented as means ± standard deviations. IQ, intelligence quotient; SSS, Subjective Socioeconomic Status Scale; CES-D, Center for Epidemiologic Studies Depression Scale; MASC, Multidimensional Anxiety Scale for Children; SDQ, Strength and Difficulties Questionnaire; APSD, Antisocial Process Screening Device; CU, callous-unemotional; BIS, Barratt Impulsivity Scale.

*p<0.05

**p<0.01.

### Conventional fMRI

**Fig 1:**
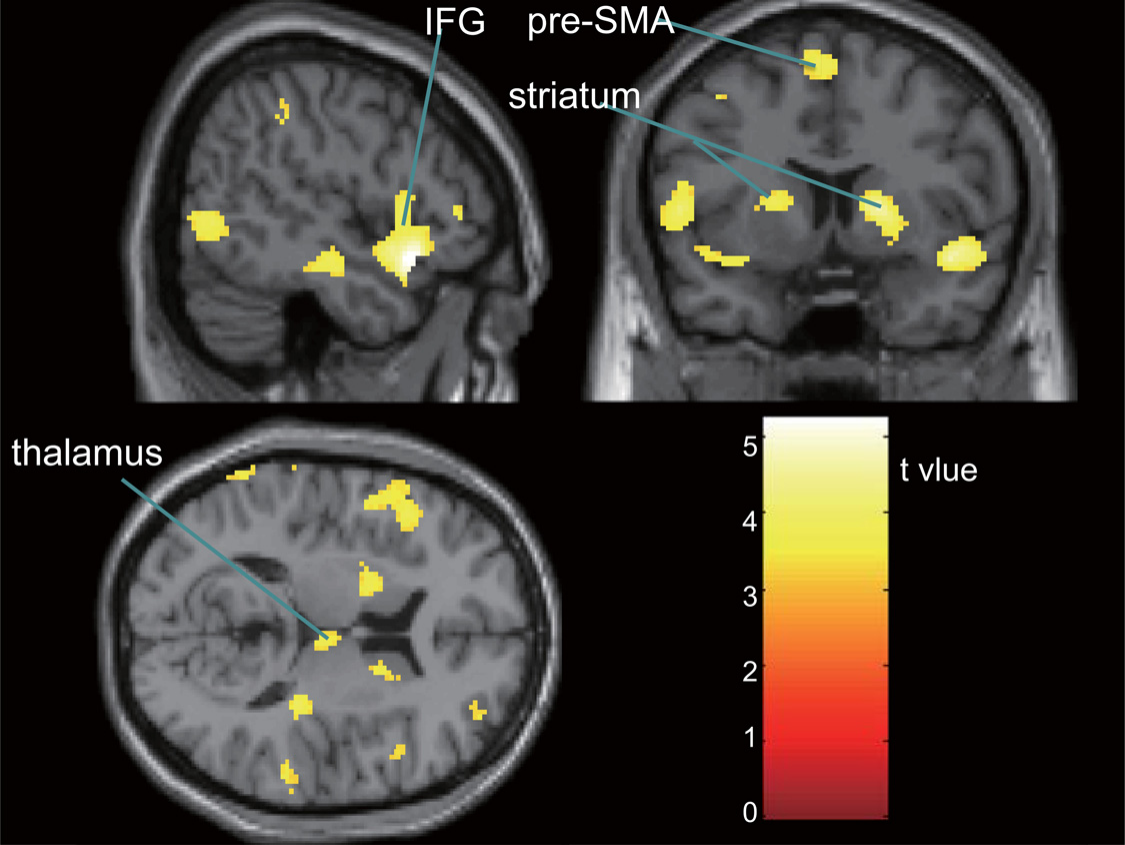
Brain regions with significantly lower activation in the conduct disorder group relative to the healthy control group during response inhibition (p< .05 [false discovery rate–corrected]). IFG, inferior frontal gyrus; pre-SMA, pre-supplementary motor area.

First-level analysis demonstrated activation in the frontal, temporal, parietal, and occipital lobes in HCs, and in the right thalamus and part of the occipital lobe in subjects with CD (both *p*< .01 [FDR-corrected]). Second-level analysis showed significantly less activation in the CD group than in the HC group, including in the bilateral IFG, middle frontal gyrus (MFG), insula, superior temporal gyrus, middle temporal gyrus, inferior temporal gyrus, striatum, inferior parietal lobule (IPL), supramarginal gyrus (SMG), middle occipital gyrus (MOG), and declive (all *p*< .05 [FDR-corrected]; Fig 1, Table 2). Brain areas with significantly lower activation in the CD group relative to the HC group also included the right thalamus and anterior cingulate cortex, and the left precentral gyrus and the pre-SMA (all *p*< .05 [FDR-corrected]; Table 2). Activation was not significantly greater in the CD group than in the HC group in any brain region.

**Table 2:** Brain regions showing significantly lower activation in the conduct disorder group relative to the healthy control group during response inhibition.

L/R	*Voxel size*	p_*FDR*_	T-value	MNI coordinates			Cerebral cortex
				x	y	z	
R	1035	0.009	5.23	50	18	-14	IFG/STG/insula
R	896	0.009	5.00	62	-18	-12	MTG/STG
R	402	0.009	4.73	18	8	6	Putamen/caudate
R	274	0.010	4.41	40	48	2	MFG/IFG
R	170	0.011	4.30	54	-72	2	ITG/MTG/MOG
R	595	0.013	4.11	58	-36	20	IPL/STG/SMG
R	94	0.016	3.89	36	-64	-30	Declive
R	40	0.017	3.76	6	34	18	Anterior cingulate
R	41	0.019	3.67	6	-18	12	Thalamus
L	971	0.009	5.13	-62	-42	30	IPL/STG/SMG
L	247	0.009	5.01	-38	-4	54	MFG/PG
L	1051	0.011	4.30	-46	22	8	IFG/ insula/STG/PG
L	286	0.011	4.26	-22	-72	-24	Declive
L	80	0.014	3.99	-66	-24	-12	MTG
L	149	0.014	4.00	-20	2	14	Putamen
L	53	0.020	3.54	-52	-52	-18	ITG
L	126	0.015	3.94	-2	6	62	pre-SMA
L	45	0.019	3.65	-30	-94	16	MOG

L, left; R, right; MNI, Montreal Neurological Institute; IFG, inferior frontal gyrus; STG, superior temporal gyrus; MTG, middle temporal gyrus; MFG, middle frontal gyrus; ITG, inferior temporal gyrus; MOG, middle occipital gyrus; IPL, inferior parietal lobule; SMG, supramarginal gyrus; PG, precentral gyrus; pre-SMA, supplementary motor area, FDR, false discovery rate.

### EC

**Fig 2:**
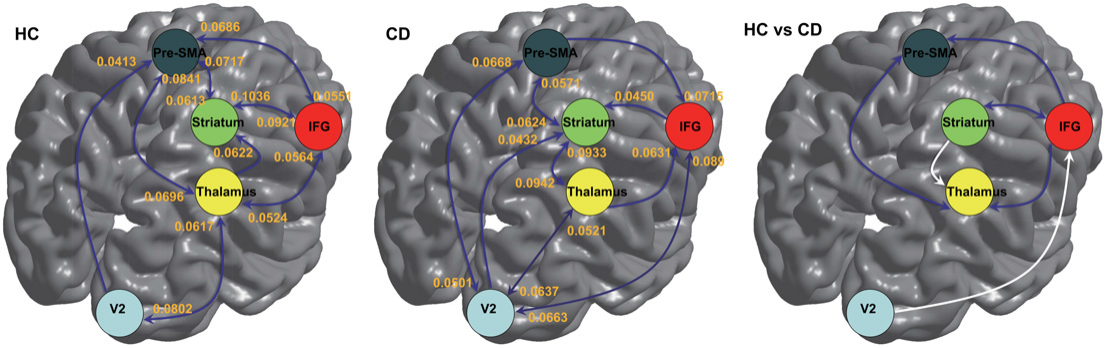
Effective connectivity within the response inhibition network in healthy controls (HC) and subjects with conduct disorder (CD). Left: Significant connectivity in the HC group; middle: significant connectivity in the CD group; right: connectivity showing significant group differences (dark blue, HC>CD; white, CD>HC). IFG, inferior frontal gyrus; pre-SMA, pre-supplementary motor area.

Significant connections at the group level (one-sample *t*-test) are shown in Fig 2. Significant connectivity between the V2 and thalamus, striatum and pre-SMA, and from V2 to the pre-SMA, from the IFG to the striatum, from the thalamus to the IFG, from the thalamus to the striatum, and from the pre-SMA to the IFG was observed in both groups (Fig 2 left and middle). However, EC from the IFG to the thalamus, from the IFG to the pre-SMA, from the striatum to the IFG, and from the pre-SMA to the thalamus was significant only in the HC group. Conversely, EC from V2 to the IFG, from V2 to the striatum, from the IFG to V2, from the striatum to the thalamus, and from the pre-SMA to V2 was significant only in the CD group (Fig 2 middle).

Two-sample *t*-tests revealed that the strength of connection between the thalamus and pre-SMA, IFG and striatum, from the IFG to the pre-SMA, and from the IFG to the thalamus was significantly greater in the HC group than in the CD group (Fig 2 right; p < .002). In contrast, EC from V2 to the IFG and from the striatum to the thalamus was significantly higher in the CD group than in the HC group (Fig 2 right; p < .002).

### Correlations between EC and behavioral scores in the CD group

**Fig 3:**
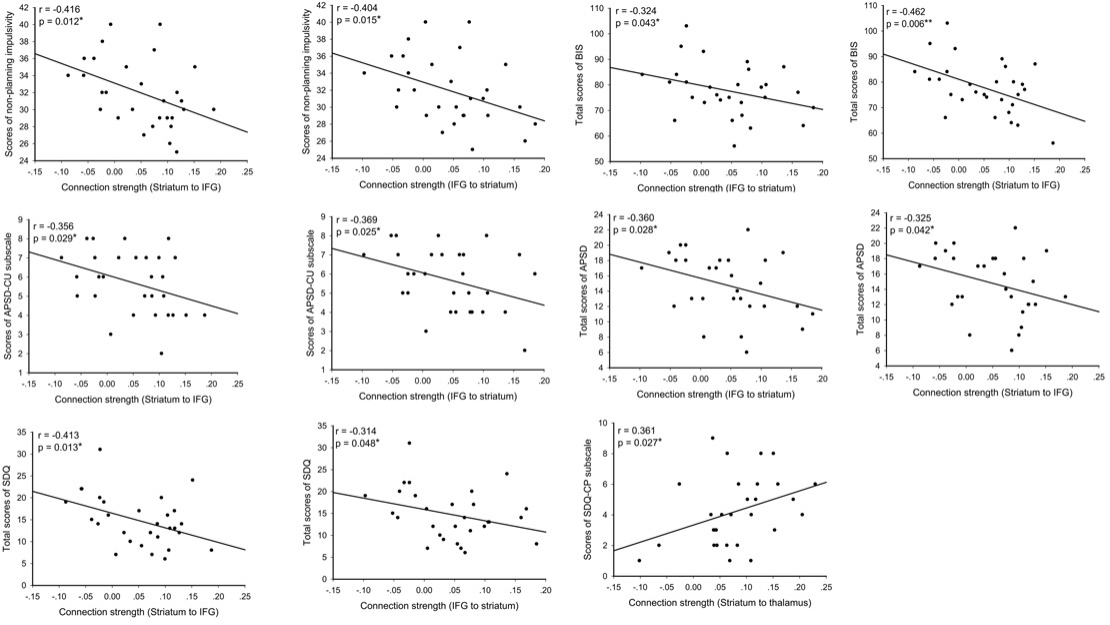
Correlations between effective connectivity and behavioral scores in the conduct disorder group (*p*< .05).

For the CD group, EC between the IFG and striatum was significantly and negatively associated with BIS total and nonplanning subscale scores, APSD total and CU subscale scores, and the SDQ total score (p < .05, uncorrected). Notably, EC from the striatum to the thalamus was correlated positively with the SDQ conduct problems subscale score in the CD group (p = .027, uncorrected) (Fig 3).

Of note, multiple regression analysis with the CU traits (APSD-CU scores) and number of conduct problem (SDQ-CP scores) as independent variables, showed significant associations between CU traits and EC from the IFG to the striatum (t = -2.124, p = .043, uncorrected), and from the striatum to the IFG (t = -2.280, p = .031, uncorrected), as well as between SDQ-CP scores and EC from the striatum to the thalamus (t = 2.042, p = .051 [marginal], uncorrected), and from the thalamus to the striatum (t = 2.611, p = .015, uncorrected), respectively.

## Discussion

The present study is the first to examine direct connectivity in the inhibition control network in subjects with CD performing a GoStop task. We found distinct interactive patterns in the inhibition control network in subjects with CD and HCs. Prominent correlations were found between behavioral scores and EC, with significant between-group differences. Conventional fMRI analysis performed before EC analysis also confirmed the typical findings reported in previous studies [30,36,37]. Thus, our study findings suggest that the impairment of inhibition control in subjects with CD is associated not only with abnormal activation in several specific brain regions, but also with aberrant direct interactions between these regions. Given that Rubia et al (2008) also performed conventional fMRI analysis of a similar task [35], whereas the present study was the first to use DCM analysis of data from subjects with CD, the following discussion focuses mainly on direct connectivity in the “stop” network.

Generally, previous studies have shown that the IFG, striatum, pre-SMA and thalamus are core regions involved in the inhibition response control network [30,31,33,34]. In DCM analysis, connection strength describes the strength and speed of influence on the target region, greater EC indicates a more rapid effect on the target region, resulting in more efficient termination of the action [38]. Thus, in the present study, the strength of EC between the IFG and the striatum, between the pre-SMA and the thalamus, from the IFG to the pre-SMA, and from the IFG to the thalamus was significant lower in CD group than that in HC group, representing slower suppression of responses and finally leading to the failure to inhibit an initiated motor response in the GoStop task [36].

Concretely, our study revealed mutually decreased interaction between the IFG and the striatum in the CD group relative to HCs, which was further and indirectly supported by the negative correlation between EC and behavioral scores in subjects with CD. Reduced activation in the dorsolateral PFC and striatum has been found in subjects with CD relative to HCs when performing the SST [35]. Recently, significantly reduced activation in the right IFG was also found in ODD relative to HCs when performing the GoStop task [39]. The IFG influenced basal ganglia circuitry indirectly *via* projection to the pre-SMA [40], which functions to reinforce wanted behaviors and suppress unwanted behaviors [41] and is involved in motor planning [40,42]. Good interaction between these regions appears to enable the successful inhibition of an initiated action [31,43], especially, stronger EC from the IFG to the striatum stopped initiated motor actions more efficiently [42]. Notably, less activation and lower EC strength (between the IFG and the striatum, and from the IFG to the pre-SMA) were also reported in impulsive control disorders (IAD) when comparing to HCs, which led to inhibition failure when performing the GoStop task [36]. This might also be applied to our results that, for the CD, lower EC lead to the disability to effectively inhibit the predominant ongoing actions.

The thalamus connects with the pre-SMA and the PFC (including the IFG) structurally and functionally [33,37,44]. Notably, no significant direct connectivity between the pre-SMA and thalamus was observed in the CD group in the present study. Thus, significantly reduced activation in the pre-SMA and IFG, along with significantly reduced strength of EC (between the pre-SMA and thalamus and from the IFG to the thalamus), might be involved in impaired motor planning in subjects with CD. This proposed relationship is supported by the significantly higher BIS nonplanning subscale scores in subjects with CD relative to HCs in our study. Interestingly, this study was the first to document significantly increased direct EC from the striatum to the thalamus in subjects with CD relative to HCs, and a positive correlation between this increased EC and SDQ conduct problem subscale scores, indicating that EC strength increases with the severity of conduct problems in subjects with CD. However, from a biochemical perspective, the striatum acts on the thalamus *via* the globus pallidus/substantia nigra [33,37]. Thus, the findings of greater EC strength in the present study should be studied further to elucidate this phenomenon.

It seems that our results of DCM analysis support the notion that when an external “stop” signal appears, the sensory input is firstly relayed to the frontal cortex through a connection from the vision cortex (V2) to the pre-SMA, and the pre-SMA exchanges information with the IFG, then the IFG sends a “stop” command to the striatum, finally the command is sent to the thalamus via some other brain regions (such as the STN, which was not significantly activated in the present study). Of note, all the connections were bidirectional (in order to communicate with each other and send the “correct” command signal) except the V2 to the pre-SMA and thalamus to the striatum. However, relative to the HC, several new connections (the V2 to the striatum, the striatum to the thalamus, between the V2 and the IFG) appeared in CD subjects, but some vital connections (between the pre-SMA and the thalamus, from the IFG to the pre-SMA, and from the IFG to the thalamus) were lacked. Importantly, all connection strength between the “stop” circuit nodes, except the one from the striatum to the thalamus, was lower than that in the HC, leading to the failure of inhibition for the CD. Besides, no phenomenon of unidirectional greater EC in one group but bidirectional greater EC strength in the other group was observed in the present study.

Of importance, the CU traits have been suggested to be one marker to classify CD [45], and one recent research has found that level of CU traits and number of conduct problems (CP) may interact, in that the influence of one may suppress the influence of another [46]. In our results, regression analysis showed that the CU traits correlated negatively with EC between the IFG and the striatum, while the number of CP was significantly positively correlated with EC between the striatum and the thalamus. Although their directions of correlation were opposite, the ECs they correlated with were different ones. Thus, we did not find significant interaction between CU traits and CP, which might be due to different tasks used in ours’ and Sebastian et al’s studies [46].

Several limitations of this study should be mentioned. First, all subjects with CD had the adolescent-onset form of this disorder; thus, the results cannot be applied directly to those with child-onset CD. Meanwhile, all the participants were males, and now there is an increasing number of literatures looking at girls with CD and mixed samples, which is why we should be cautious that our results could not be simply generalized to females or mixed subjects with CD. Second, just as mentioned by Daunizeau et al [47], one of the main difficulties of stochastic DCM is inherent in hemodynamic modeling, as the causal impact of neural states on observed BOLD signals is delayed, which turned out to be more problematic than for deterministic DCM, and then limited the ability of stochastic DCM to recover the neural state dynamics and the network structure. Also, no special steps were performed to resolve this problem when we performed the DCM analysis, which might influence the power of our results. Third, nether data in terms of response time, nor information about the effect of response time on EC strength was reported in our study, which might be another limitation for our present research. To compensate the lack of the response time files, we performed correlations between scale scores and EC, instead. Actually, Dougherty et al (2003) has used the GoStop task in the CD subjects, and the results showed a lower inhibited response rates to stop trials for the CD group than for the healthy control, suggesting the present task paradigm could be a useful approach to access the lack of inhibition control in the CD [26].

In sum, this study is the first to use a DCM to examine CD, and significantly different patterns of EC in the inhibition control network were found between the CD and HC groups. Our results suggest for the first time that the failure of inhibition control in subjects with CD might be associated with aberrant connectivity of the frontal–basal ganglia pathways, especially between the IFG and striatum.

## Methods

### Sample

A total of 32males with CD aged 14–17 years were recruited from outpatient clinics affiliated with the Second Xiangya Hospital of Central South University in Changsha, Hunan, China. Detailed information on participant recruitment has been published previously [28]. The HC group (no history of CD/ODD and no current psychiatric illness) comprised40 age-, gender-, and IQ-matched volunteers recruited from a regular school in the same city. Three subjects with CD were excluded from further analyses due to excessive head motion (≥2.5mm or ≥2.5°); 29 subjects with CD were thus included in the final analysis. All participants with CD were treatment naïve and fulfilled the DSM-IV criteria for adolescent-onset CD [1].The study was conducted in accordance with the Declaration of Helsinki and was approved by the Ethics Committee of the Second Xiangya Hospital of Central South University (No: CSMC-2009S167). All subjects and their parents were aware of the purpose of the study and provided written informed consent. Exclusion criteria included IQ< 80, according to the Chinese revision of the Wechsler Intelligence Scale for Children [48].

Two well-trained psychiatrists independently screened all participants for CD, ADHD, ODD, and other psychiatric and emotional disorders; pervasive developmental and chronic neurological disorders, Tourette’s syndrome, post-traumatic stress disorder, and obsessive compulsive disorder; persistent headache; and alcohol or substance abuse based on the Structured Clinical Interview for the DSM-IV-TR Axis I Disorders, Patient Edition [49]. All the subjects were right-handed according to the Edinburgh Handedness Inventory [50]. The Chinese version of the Center for Epidemiologic Studies Depression Scale [51] and the Multidimensional Anxiety Scale for Children [52] were used to assess the severity of individuals’ depression and anxiety, respectively. The Chinese version of the Subjective Socioeconomic Status Scale [28] and the Strength and Difficulties Questionnaire (SDQ) [53] were used to assess participants’ socioeconomic status and internalizing and externalizing problems, respectively. The Antisocial Process Screening Device (APSD) [54] was used to assess subjects’ callous-unemotional (CU) traits, the presence of which has been found to be useful for the distinction of CD types [3].The Barratt Impulsivity Scale (BIS) [55] was used to assess impulsivity and aggression, prominent features of CD [1].

### GoStop task

**Fig 4:**
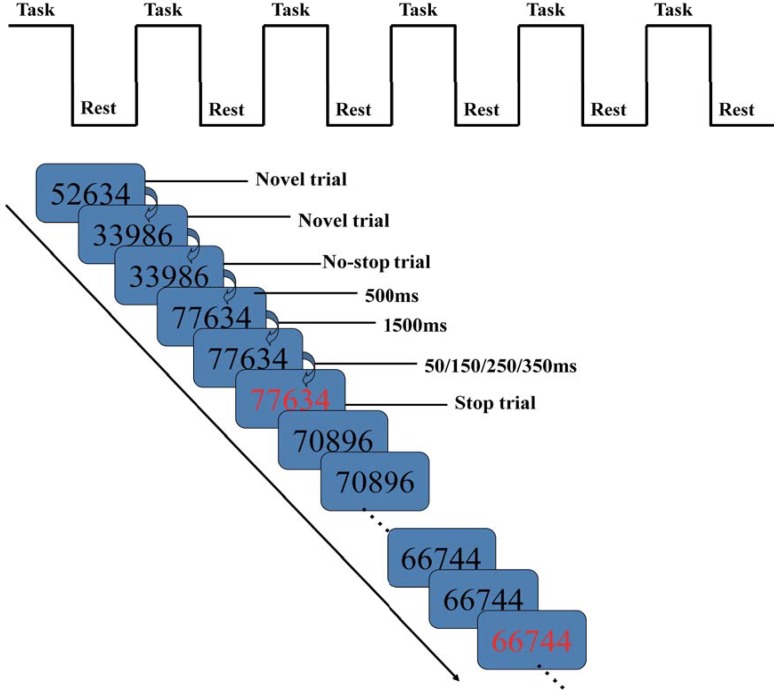
The GoStop task paradigm used in this study.

The GoStop task is a paradigm used to measure the capacity to inhibit an initiated predominant response [27], which has been used to check the abnormal brain activation of ODD [39] and the internet addiction disorder (IAD) [36].It requires the participant to watch a series of five-digit black numbers appearing against a white background, responding when a target "go" signal appears and withholding response when a “stop” signal or non-target stimulus appears. The task is comprised of no-stop, stop, and novel trial types (Fig 4). In the no-stop trial, the “go” signal is a number identical to the previous number presented in black; the participant must press a button accurately and in a timely manner, before the number disappears from the screen. In the stop trial, the “stop” signal is a stimulus matching the preceding one but changing from black to red unpredictably, at some specified asynchrony (50, 150, 250, or 350 ms) after stimulus onset, requiring the participant to try to withhold a response. In the current study, the intervals of the stimulus color change occur with equal probability. In the novel trial, randomly generated non-matching numbers are presented in black [27].

A block design was used for the GoStop task in this study (Fig 4) [36]. It began with a 70-s task block, followed by a 20-s rest block. During the rest block, the word “rest” was displayed at the center of the screen, which was then followed by another 70-s task block. This sequence of task and rest blocks were repeated six times, with each scanning session lasting 9 min. For each task block, stimuli were presented on the screen for 500 ms each, separated by a 1500-ms off period.

### Neuroimaging methods

#### Image acquisition and preprocessing

fMRI data (repetition time/echo time = 2000/30 ms, slice thickness = 4 mm, number of slices = 36, matrix size = 64 × 64, field of view = 240 × 240 mm, flip angle = 90°) were acquired during the task using a PHILIPS Achieva 3.0T whole-body scanner(Amsterdam, Netherlands) at Second Xiangya Hospital. The fMRI data were preprocessed using statistical parametric mapping (SPM12, http://www.fil.ion.ucl.ac.uk/spm). First, the images were realigned to the first image obtained in each session using six-parameter rigid body transformation. Second, all images were spatially normalized to a Montreal Neurological Institute template with affine registration, followed by nonlinear transformation, using a voxel size of 2 × 2 × 2 mm. Finally, the data were smoothed with an 8-mm full width at half maximum Gaussian kernel [36].

#### Statistical parametric mapping

A general linear model (GLM) was used to model subject-specific responses, and onsets of accurate no-stop, stop, and novel trials were modeled after convolution with a hemodynamic canonical basis function. Six motion parameters were included to model movement-correlated effects [36]. In the first-level (within-subject) analyses, the contrast “stop>baseline” was determined, enabling identification of brain regions that were significantly activated or deactivated when subjects tried to inhibit responses to stop trials [36]. The resulting contrast images were included in the second-level (between-subject) analyses. One-sample *t*-tests were used to show brain activation during response inhibition for each group, and two-sample *t*-tests were performed to examine group differences in brain activation when performing the task. Finally, group differences were corrected for multiple comparisons using the false discovery rate (FDR) correction at *p*< .05.

#### Dynamic causal modeling

EC analysis was performed using the stochastic DCM12 [36]. This model allows for endogenous or random fluctuations in unobserved (hidden) neuronal and physiological states, known technically as system or state noise; it differs in this way from a conventional deterministic DCM, and provides more accurate parameter estimates [38]. Following Li et al (2014), the three task inputs (from no-stop, stop, and novel trials) were concatenated to one input for the DCM analysis to reduce model complexity. Finally, the contrast “task > rest” was determined for each individual [36].

Based on the results of group analysis, as well as those of previous fMRI studies of response inhibition, we defined four regions of interest (ROIs): the right IFG, right striatum, right thalamus, and left pre-SMA (Fig 2). A fifth region or node of visual cortex (V2) was added to the model because the activity within the motor system can be assumed to be driven by the visual system when responding to visual stimuli. As in Li et al (2014), no significant activation of the STN was observed during task performance; thus, we did not include this region in the final DCM analysis. Subject-specific ROIs (radius = 6 mm) were centered on the peaks of SPMs testing for the contrast ‘‘task > rest” [36]; time series were then extracted for these ROIs. For some subjects, the locations of the ROIs were adjusted slightly to make sure they located within the same anatomical gyrus as the group maximum [36].

For the DCM analysis, a fully connected model was first constructed and subject-specific DCMs were fully and reciprocally connected (resulting in 20 connections among five nodes). No bilinear or modulatory effect was modeled in this study, as our main interest was the detection of group differences in EC [36]. In other words, we estimated the average connectivity under the task set of response inhibition, assuming that endogenous fluctuations in neuronal activity (state noise) would model condition-specific responses [36]. Generalized filtering was used to invert the fully connected model for each individual [56].The optimal model pooling all subjects was identified using a network discovery scheme [38,57,58], and the full connected model was just the optimal one. Finally, subject-specific parameter estimates (posterior means) under the optimal model were included in the second-level(between-subject) analysis using classical random effects, which enabled the analysis of findings from subject-specific DCMs using classical statistics (*t*-tests with Bonferroni correction for multiple comparisons, *p*< .05) at the group level [36].

## Supporting Information

S1 File Distribution of behavioral scales scores. (XLSX)
